# Medical student remote eConsult participation during the COVID-19 pandemic

**DOI:** 10.1186/s12909-021-02562-6

**Published:** 2021-02-22

**Authors:** Adam R. Kopp, Sharon Rikin, Todd Cassese, Matthew A. Berger, Amanda C. Raff, Inessa Gendlina

**Affiliations:** 1 Albert Einstein College of Medicine, 1300 Morris Park Avenue, Bronx, NY 10461 USA; 2 Department of Medicine, Division of General Internal Medicine, Albert Einstein College of Medicine and Montefiore Medical Center, 1300 Morris Park Avenue, Bronx, NY 10461 USA; 3 Department of Medicine, Division of Hospital Medicine, Albert Einstein College of Medicine and Montefiore Medical Center, 1300 Morris Park Avenue, Bronx, NY 10461 USA; 4 Department of Medicine, Division of Nephrology, Albert Einstein College of Medicine and Montefiore Medical Center, 1300 Morris Park Avenue, Bronx, NY 10461 USA; 5 Department of Medicine, Division of Infectious Diseases, Albert Einstein College of Medicine and Montefiore Medical Center, 1300 Morris Park Avenue, Bronx, NY 10461 USA

**Keywords:** COVID-19, eConsults, Medical education, Medical student, Telemedicine, Curriculum development

## Abstract

**Background:**

Undergraduate medical education was severely impacted by the COVID-19 pandemic. As traditional clinical rotations were suspended, medical students quickly began alternative, novel educational experiences. Third-year medical students at an academic medical center were given the opportunity to join inpatient eConsult teams within the department of medicine. This study describes the development and implementation of this program as well as the experiences of student and faculty participants.

**Methods:**

Student eConsult participation was rapidly developed and implemented within medical subspecialty teams in either infectious diseases (ID) or nephrology. Twelve third-year medical students and 15 subspecialty attendings participated in this program during an eight-week period from April 6 through May 29, 2020. Breadth of student clinical experience was assessed via review of clinical documentation and surveys. Participating students and attending physicians completed surveys to reflect upon their impressions of the program. Surveys were returned by nine students and eight faculty members. Survey responses were summarized with descriptive statistics.

**Results:**

Over an eight-week period, student consultants wrote 126 notes on 100 patients; 74 of these patients (74%) were hospitalized with COVID-19. Student experiences were largely positive with most strongly agreeing that attendings promoted interactive and engaged learning (*N* = 8 of 8, 100%), that the experience helped to expand their knowledge about consultant roles (*N* = 6, 75%), and that they would participate in a remote eConsult program again if given the opportunity (*N* = 6, 75%). Faculty also were largely positive about the experience with most agreeing or strongly agreeing with the importance of teaching medical students about telehealth (*N* = 7 of 8, 88%) and eConsults (*N* = 6, 75%). In narrative responses, students and faculty agreed that teaching was a strength of the program whereas lack of in-person contact was a challenge.

**Conclusions:**

Rapid development of an inpatient eConsult-based educational experience for third-year medical students was feasible and successful. Student-consultants saw a range of pathology including COVID-19 and related complications. Students were satisfied with the program. They were able to develop a strong relationship with attendings while learning about the role of a consultant. Faculty agreed with the importance of teaching students about telehealth and eConsults specifically.

**Supplementary Information:**

The online version contains supplementary material available at 10.1186/s12909-021-02562-6.

## Background

The coronavirus disease 2019 (COVID-19) pandemic has resulted in greater than 95 million confirmed cases worldwide and nearly 24 million confirmed cases in the United States as of January 18, 2021 [1]. New York City was one of the earliest epicenters in the US, with the first case of community transmission reported in early-March 2020. Healthcare systems in NYC underwent rapid reorganization in order to provide medical care to surging numbers of patients with COVID-19 [2]. At academic medical centers nationally, medical students were suspended from all clinical activities on March 15, 2020 [3]. Immediately following the shut-down, student and faculty leaders developed initiatives to safeguard the health of students and the community, provide access to novel medical educational experiences, and address emerging needs of affiliated health care systems.

Electronic consultations (eConsults) represent one of several telecommunication options that are widely implemented in primary care and outpatient medicine [4, 5]. During the COVID-19 pandemic, Montefiore Medical Center, a large academic medical center in the Bronx, NY developed and implemented a novel inpatient eConsult model for subspecialty consultation. Modeled after an ongoing outpatient eConsult program [6], this inpatient eConsult program allowed primary and specialist teams to communicate asynchronously within the electronic medical record (EMR). This allows for inter-provider consultation in situations when limited patient contact is needed for health care provider and public health safety and provides enhanced access to specialty expertise without in-person interaction. Early during the COVID-19 pandemic, the inpatient eConsult program allowed rapid response from specialty experts while preserving precious personal protective equipment (PPE).

Medical students are involved in consultative specialty and subspecialty services as part of their clinical rotations, yet few studies describe student-consultant experience and overall education benefits [7, 8]. Additionally, there is limited literature regarding the specific roles and experience of medical students as consultants in internal medicine subspecialties or as participants in telemedicine. The purpose of this study is to describe the integration of an eConsult service at an undergraduate medical education program during a pandemic. This is the first study of medical student participation in an inpatient eConsult program in response to COVID-19.

## Methods

### Study design, setting and participants

An inpatient eConsult program was developed and implemented at Montefiore Medical Center in the Bronx, NY in response to the COVID-19 pandemic. Inpatient eConsults were made available on March 7, 2020. The inpatient eConsult workflow was developed from an existing ambulatory eConsult program. Requests for specialty eConsults were orderable in the EMR and monitored by eConsult specialist teams. eConsult requests were answered as fully documented Initial or Follow-up consult notes. Third-year medical students remotely assisted attending consultants in the infectious diseases (ID) and nephrology divisions. Students had authorization for password protected use of the EMR for clinical care and note writing consistent with usual participation by students in clinical care. Notes written by trainees required attending review and attestation as with traditional consult service documentation.

### Program logistics

Student participation in the inpatient eConsult program began on April 6, 2020 with a two-week pilot period involving a single student on an ID teaching team. After this period, other students were invited to apply to an expanded version of the program. Interested students were vetted by program leadership and those with a strong academic record were selected by lottery. Students attended a brief orientation via Zoom and were provided with the contact information of their assigned attending. Students communicated with attendings via telephone and e-mail to go over consult assignments and to present patients. A total of ten students were recruited to participate in ID eConsult services. During this same period, an additional student joined a nephrology eConsult service. During this period all students continued to participate in their required virtual clerkship experiences and shelf exams. Students were asked to participate for at least 1 week and were able to extend their enrollment for as long as the program was operational. Student terms therefore varied from 1 week to more than 4 weeks on the basis of individual student preferences.

Overall, there were 12 student participants in the eConsult volunteer program. Notes were written by 11 of 12 students in the EMR. All student notes written over the course of the program were reviewed. Student-author (ARK) was the pilot student and was excluded from participation in the program evaluation survey. This study was approved by the Einstein Institutional Review Board.

### Survey development and participation

Student and faculty survey instruments were created to elicit participant reflections and assess their satisfaction with this experience. Evaluated domains included knowledge, learning, patient management, supervision, and satisfaction. The student survey consisted of 13 multiple-choice questions, nine Likert scale questions, one forced ranked-choice question, and eight short answer questions (Additional file 1). The faculty survey consisted of nine Likert scale questions and four short answer questions (Additional file 2).

### Review of data elements

All notes written by participating medical students on the consult teams during the study period were reviewed for reason for consult, and whether it was an initial or follow-up consult. In order to identify the breadth of topics covered, notes were categorized to determine whether a given consult involved a question primarily about COVID-19, about another topic or complication in a patient hospitalized with COVID-19, or about a patient admitted with another, non-COVID-19, condition.

### Data analysis

Review of student notes was used to identify reason for consultation. Quantitative survey results were analyzed using Qualtrics and Microsoft Excel. Narrative question responses within surveys were reviewed and grouped based on predefined domains to provide a qualitative and descriptive account of participant experience.

## Results

### Student characteristics

A total of 12 students participated in the inpatient eConsult program. Nine of 11 eligible student participants completed a brief survey describing their experience with the eConsult program. Two students reported that they had never spent any time on an inpatient Internal Medicine consultation team, whereas the remaining seven students had spent at least 1 week on such a team. Despite familiarity with inpatient consults, five students had no experience providing care via telehealth. Three students reported between 1 week and 1 month of telehealth experience, and one student reported more than 1 month. While performing eConsults, five students were located at an off-campus location allowing secure and confidential communication while four students were located on-campus but not in the hospital. No students participated for less than 1 week, and five of nine students chose to participate for 4 weeks or more. Eleven students wrote 126 notes on 100 unique patients. During their time on the eConsult services, students wrote a mean of 11.5 notes on a mean of 9.1 patients (Table 1).

**Table 1:** Characteristics of a novel inpatient eConsult experience

**Program Characteristics** *Participants:*	
Students (Survey Respondents) *n* = 11	*N* = 9 (81%)
Faculty (Survey Respondents) *n* = 15	*N* = 8 (53%)
*Students in Medical subspecialties:*	
Infectious Diseases	*N* = 11
Nephrology	*N* = 1
**Student Characteristics**	
*Previous in-person inpatient consultation experience:*	
None	*N* = 2
Less than 1 week	*N* = 0
Between 1 week and 1 month	*N* = 6
More than 1 month	*N* = 1
*Previous telehealth experience:*	
None	*N* = 5
Less than 1 week	*N* = 0
Between 1 week and 1 month	*N* = 3
More than 1 month	*N* = 1
*Location during eConsults:*	
Off-campus secure location	*N* = 5
On-campus, non-clinical space	*N* = 4
*Duration of eConsult participation:*	
Less than 1 week	*N* = 0
1 week	*N* = 1
2 weeks	*N* = 1
3 weeks	*N* = 2
4 weeks	*N* = 3
5 weeks	*N* = 2
More than 5 weeks	*N* = 0
**Consult Characteristics:**	
Total Notes	*N* = 126
Unique Patients	*N* = 100
Notes/Student	11.5
Patients/Student	9.1

### Consult content

Of the 100 patients followed by students, 74 were admitted for COVID-19 of whom 46 had consults related to primary acute COVID-19 and 28 had consults regarding complications related to COVID-19. The 26 COVID-19 negative patients were evaluated for other medical conditions. Students participated in care for three patients requiring renal consultation and 97 patients requiring ID consultation.

**Fig. 1:**
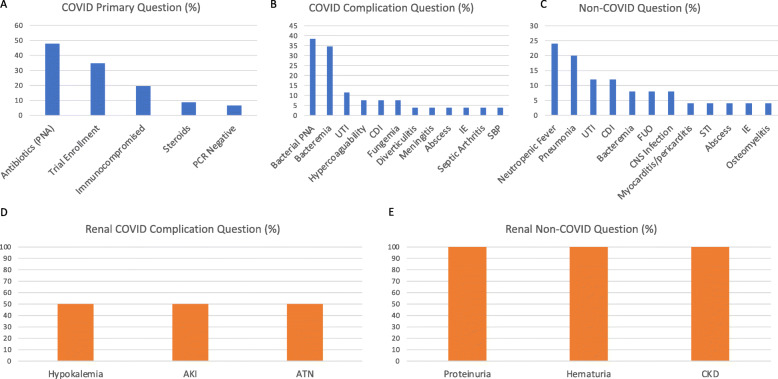
Content of inpatient eConsults seen by medical students. Student consult notes were reviewed to determine whether the consult question was related to a primary COVID-19 problem, a complication in a patient hospitalized with COVID-19, or a medical condition in a patient without COVID-19. **a**-**e** Unique topics seen by student consultants as COVID primary (*N* = 46, ID), COVID complication (*N* = 26, ID; *N* = 2, renal), or non-COVID (*N* = 25, ID; *N* = 1, renal) cases are reported as the percent of subspecialty notes addressing that topic (blue bars = ID consults, orange bars = renal consults). As patients could have more than one medical condition addressed in a consult, numbers are not additive

Among the COVID-19 consult requests to ID, the most common reason for consult was about the need for antibiotics to treat superimposed bacterial infection (addressed in 48% of patients). Other common questions were about clinical trial enrollment or the use of experimental treatments for COVID-19 (35%) and about treatment considerations in immunocompromised patients (20%) (Fig. 1a).

There was a range of pathology seen in the eConsult requests related to complications in patients hospitalized with COVID-19. The three most common questions to ID were regarding secondary infections, specifically pneumonia (38%), bacteremia (34%), and urinary tract infection (UTI) (12%) (Fig. 1b). The three most common questions to nephrology were hypokalemia (50%), acute kidney injury (AKI) (50%), and acute tubular necrosis (50%) (Fig. 1d).

For patients who did not have COVID-19, the most frequent ID problems addressed were neutropenic fever (24%), pneumonia (20%), and UTI (12%) (Fig. 1c). On the renal eConsult team, one patient was evaluated for proteinuria, hematuria, and chronic kidney disease (CKD) (Fig. 1e).

Follow-up notes comprised 44% of the total notes written by students. For the ID eConsults, the three most frequent reasons for follow-up were antibiotics (addressed in 44% of follow-up notes), new microbiology result (28%), and new or persistent fevers (25%). For the renal eConsults, the most frequent reasons for follow-up were ongoing work-up (67%) and electrolyte abnormalities (33%).

### Student experiences

**Fig. 2:**
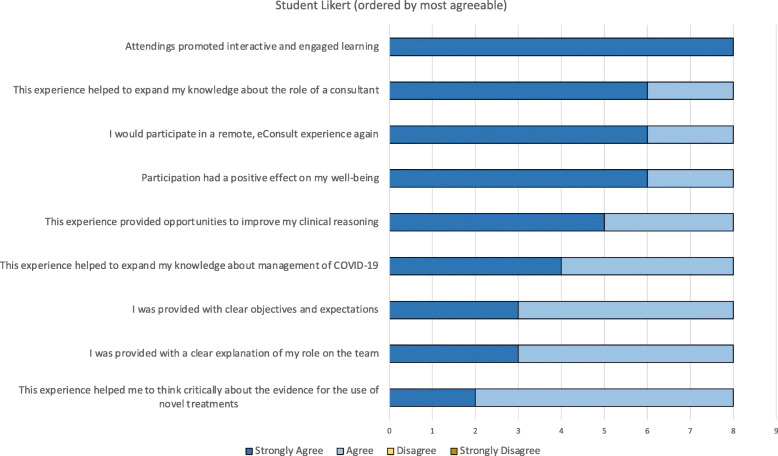
Student appraisal of eConsult experience. Students were asked to respond to nine statements with a four-point Likert scale. Statements are listed here ordered by the number of students (*N* = 8) who Strongly Agree

Of the nine students who began the survey instrument, eight completed the Likert section. All eight students strongly agreed with the statement “Attendings promoted interactive and engaged learning.” The other statements with the highest proportion of students strongly agreeing were “This experience helped to expand my knowledge about the role of a consultant” (*N* = 6 of 8, 75% Strongly Agree), “I would participate in a remote, eConsult experience again” (*N* = 6, 75%), and “Participation had a positive effect on my well-being” (*N* = 6, 75%). Only two students strongly agreed with the statement “This experience helped me to think critically about the evidence for the use of novel treatments.” The other statements with the lowest proportion of students strongly agreeing were “I was provided with clear objectives and expectations” (*N* = 3, 38%) and “I was provided with a clear explanation of my role on the team” (*N* = 3, 38%). Of note, all eight students answered with Strongly Agree or Agree for each of the nine Likert queries, indicating an overall positive impression of the program (Fig. 2).

**Fig. 3:**
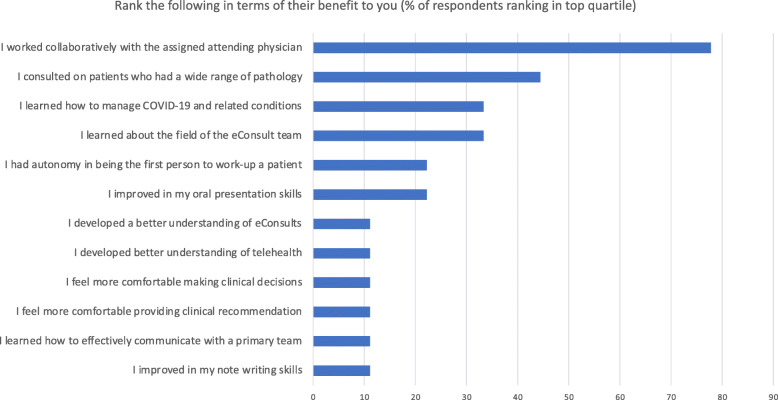
Student perception of eConsult program benefits. Students were provided with a forced-rank choice question comprising 12 items and were asked to rank these from most beneficial to least beneficial. Statements were scored based on the proportion of students (*N* = 9) ranking a given item in the top quartile (ranks 1–3)

The survey included a forced-choice question in which students ranked a series of 12 statements about their experience from most beneficial to least beneficial. All nine of the survey respondents completed this section. These statements were analyzed based on the proportion of students ranking them in the top quartile of most beneficial (i.e., spots 1–3). The highest rating statement was “I worked collaboratively with the assigned attending physician” (*N* = 7 of 9, 78% ranking in the top quartile). Six students ranked this as the most beneficial statement overall. Other statements that were ranked highly were “I consulted on patients who had a wide range of pathology” (*N* = 4, 44%) “I learned how to manage COVID-19 and related conditions” (*N* = 3, 33%), and “I learned about the field of the eConsult team” (*N* = 3, 33%) (Fig. 3).

Six of the 12 statements were ranked in the top quartile of benefit by only a single student. Among these six, the three with the lowest overall ranking by students were “I learned how to effectively communicate with a primary team,” “I developed a better understanding of telehealth,” and “I feel more comfortable making clinical decisions” (Fig. 3).

### Faculty experiences

**Fig. 4:**
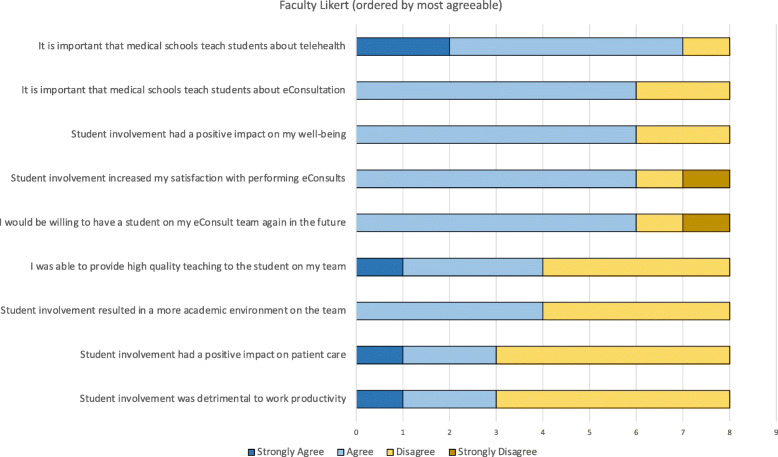
Faculty appraisal of eConsult experience. Faculty were asked to respond to nine statements with a four-point Likert scale. Statements are listed here ordered by the number of faculty (*N* = 8) who Strongly Agree or Agree

Eight out of 15 eligible faculty members who led eConsult teams with student members replied to the faculty survey. The statement with the highest number of faculty responding with Strongly Agree or Agree was “It is important that medical schools teach students about telehealth” (*N* = 7 of 8, 88% Strongly Agree or Agree). Additional highly ranked statements were “It is important that medical schools teach students about eConsultation” (*N* = 6, 75%) and “Student involvement had a positive impact on my well-being” (*N* = 6, 75%) (Fig. 4).

Among the statements that had the highest proportion of faculty responding with Strongly Disagree or Disagree were “Students involvement had a positive impact on patient care” (*N* = 5, 63% Strongly Disagree or Disagree) and “Student involvement resulted in a more academic environment on the team” (*N* = 4, 50%). Furthermore, five faculty disagreed with the statement “Student involvement was detrimental to work productivity,” however, it should be noted that disagreeing with this particular statement represents a positive impression of the program (Fig. 4).

### Free-response thematic analysis

Students and faculty each identified teaching as a strength of the program. Students noted the benefit of working closely with individual attendings. Students likewise found the return to patient care a positive aspect of eConsults, and one student wrote, “[one of the greatest strengths was] allowing students to participate in caring for patients when we could not otherwise have an in-person experience.” Another student noted that the volume of patients they saw exceeded what they had previously been exposed to on typical inpatient units (Table 2). Finally, students enjoyed learning about the different subspecialties (i.e., ID and nephrology). One faculty member highlighted the benefit of teaching students again after a period of solitary work during the COVID-19 pandemic.

**Table 2:** Thematic synopsis of student and faculty narrative responses

Theme	Student	Faculty
**Teaching**		
Positive	• Flexible scheduling with individualized interaction and learning • Ability to work closely with an attending • Increased comfort with giving basic ID recommendations • Improved oral presentation skills	• One-on-one teaching • Joy of having students back on the teaching service • Phone teaching fairly comparable to in person rounds • Opportunity to teach students who otherwise might not have benefitted.
Negative	• Scheduling issues due to attending workload	• Without a team, there was no ability for students to give short formal presentations to other team members. • Narrow subject matter.
**Mentoring**		
Positive	• Nurturing relationship with the attending.	
Negative		• Lack of in-person contact. • Time spent in discussion with the student impacted time for other learners.
**Patient Care**		
Positive	• Ability to follow increased number of patients. Opportunity for students to participate in patient care despite suspended clinical rotations. • Contribution to alleviating the burden on the consult team.	• Provided students with exposure to patient care during the pandemic from a safe vantage point
Negative	• The inability to conduct a physical exam or speak with patient • Variance in eConsult patient load • Limited communication with other teams	• Few eConsult patients at end of pandemic surge
**Communication**		
Negative	• Slower communication when not working together in person. • Lag time for questions to be answered. • Limited communication with primary care team	

Both students and faculty noted that lack of in-person contact was a weakness of the program (Table 2). Students were disappointed in not being able to have in-person contact both with their eConsult team and with patients (e.g., “not being able to do a physical exam”). For students, they also noted difficulty in communicating with other teams outside of the patients’ charts. One faculty found that, “the phone format cut into good teaching somewhat.” Another challenge encountered by both students and faculty was a lack of cases as regional COVID-19 cases waned towards the end of the eConsult period. Furthermore, some of the cases seen as eConsults were thought to be overly repetitive and formulaic for learners (Table 2).

The recommendations offered for the program were quite diverse and therefore not amenable to thematic analysis. Suggestions offered by students included pairing students with a single attending, having a more formalized orientation and set of expectations, and giving additional feedback on notes. The recommendations offered by faculty included expanding eConsults to a greater number of non-COVID patients, allowing for virtual meetings with the entire team rather than one-on-one phone calls with students and attendings, and limiting the experience to a single week.

## Discussion

The COVID-19 pandemic continues to disrupt nearly every facet of daily life, and undergraduate medical education has not escaped its reach [9–12]. To provide ongoing clinical involvement for medical students who were removed from direct patient-care rotations, a novel curricular opportunity was developed wherein students remotely joined ID and nephrology eConsult teams.

For most of the students who participated in the inpatient eConsult program, this was their first experience providing care via telehealth. Only a small number of studies have looked at medical student engagement in telemedicine initiatives, limited to underserved communities [4, 13–16]. The COVID-19 pandemic has emphasized and led to the expansion of telehealth in medical care delivery. An essential component of its adoption in medical education is teaching medical students about telehealth and including them in its dissemination [17].

During the course of the study, academic medical centers across the world likewise grappled with how to adapt medical education to the pandemic [9–12, 18–21]. A study by Su et al. describes medical student participation in ambulatory dermatology eConsults [18]. Their report highlights benefits of eConsults that include communicating as a consultant to referring providers and seeing a variety of conditions. In addition to this example of asynchronous eConsults, other studies have explored synchronous telehealth activities for medical students. In one study, third-year clerkship students participated in a weekly telehealth module and were overall satisfied with the program feedback received from their attendings [19]. In another study, medical and pharmacy students engaged in outreach to patients at risk for delays in care, demonstrating the feasibility of meaningful interprofessional education in a time of global pandemic [20]. While these reports describe novel telehealth opportunities developed in response to the pandemic, academic medical centers in the US and Europe quickly developed educational opportunities that expanded the work force by deploying students in clinical settings as temporary residents, ventilator therapy assistants and nursing assistants [21]. No studies to date describe participation of medical students specifically in inpatient eConsult programs.

In this study, a student inpatient eConsult program was both feasible and well received by learners and teachers. Strengths of the program from the student perspective included the interactive learning with frequent discussions between students and attendings, expanded knowledge of the role of consultants in patient care, and a positive effect on overall well-being for both students and faculty. While there was concern from participating faculty that the widespread prevalence of COVID-19 meant that medical students would not see a sufficient breadth of pathology, student responses and review of student notes show that this was not the case. Students consulted on a wide range of foundational topics in ID and nephrology. As such, there will be broad flexibility in tailoring an eConsult experience to meet curricular requirements.

Faculty agreed with the importance of teaching medical students about telehealth. They were, however, less uniformly positive in their appraisal of the program. Half of all faculty respondents said they were not able to provide high quality teaching to the student on their team, and that student involvement did not contribute to a more academic environment or improve patient care. Such responses may have been due to overwhelming demands on physicians during the COVID-19 surge with unprecedented numbers of eConsults addressed daily and the relative few of those patients assigned to medical students. Nevertheless, the majority of attendings indicated a willingness to have students on their eConsult teams in the future.

There are several reasons why faculty and students may have felt differently about the experience. First, it is likely that students appreciated the opportunity for individualized engagement whereas busy faculty members may have been challenged by the volume, scope and urgency of clinical care demands. Second, students may not have had prior consult experience with which to compare these experiences, whereas most attendings did. Third, the resilience of medical student learners has been previously described in settings of natural disasters and disaster relief efforts [22–24]. In their suggestions on how to improve the program, faculty differentiated working with students one-on-one and preferred teaching in the context of a larger academic team of trainees. When placing students on eConsult teams in the future, it may be beneficial to build out larger team collaborations to amplify team-based teaching.

The surveys identified several other ways in which this program might be improved going forward. Many students reported dissatisfaction with not being able to interview patients or perform a physical exam during the eConsult. Students also expressed a desire for a more robust orientation and delineation of expectations and goals. Given that the absence of patient contact is an essential element of eConsults, it is likely that these findings are related. Developing a robust curriculum which includes an orientation and syllabus that describes the rationale and motivations of eConsults will help to ameliorate student concerns with the format. As technologies infrastructure improves, it will be possible to add video capabilities to the eConsult experience. It should be noted that certain conditions are not amenable to distance care and cannot be covered in an eConsult curriculum, such as dialysis care that requires in-person management.

Limitations of this initiative include the size and scope of this pilot as it was implemented in a specific urgent circumstance. Future studies would benefit from a larger sample size and participation across multiple institutions. Furthermore, this study may be influenced by selection bias. As the students were highly motivated volunteers with strong academic records, it is possible that their experiences are not generalizable to the entire medical student body. Fidelity of the program would need to be evaluated in a large medical student class including a wide range of learners. Finally, given the decision to remove students from inpatient rotations during the early phases of the pandemic, participating students did not have traditional access to hospital opportunities. The value of such a program and the willingness of students to participate may vary when in-person patient care rotations are possible. One study conducted prior to the pandemic showed that medical students intended to practice telehealth in the future and were interested in continuing telehealth courses [25].

## Conclusions

Overall, this study demonstrates that remote inpatient eConsults provided a robust and satisfying educational experience for medical students who were unable to work in the hospital during the COVID-19 pandemic. This program was rapidly implemented through collaborative efforts of the medical school, information technology and EMR support, and departmental leadership, all working to meet the rising needs of the healthcare system. Additionally, it offers a framework for teaching students about the field of eConsults which is emerging as an important component of patient care that is expected to be an enduring aspect of health care delivery moving forward. As such, student eConsults represent a valuable curricular option in both times of emergency and normalcy, to appropriately address the educational domains of knowledge, learning, patient management, supervision, and satisfaction.

## Supplementary Information

**Additional file 1.** Student Survey.

**Additional file 2.** Faculty Survey.

## Data Availability

The datasets used and/or analyzed during the current study are available from the corresponding author on reasonable request.

## Abbreviations

PPE: Personal protective equipment
ID: Infectious diseases
COVID-19: Coronavirus disease 2019
EMR: Electronic medical record
eConsults: Electronic consults
