# Supplementary material for: Medical student remote eConsult participation during the COVID-19 pandemic
Source: BMC Med Educ. 2021 Feb 22;21:120. doi: 10.1186/s12909-021-02562-6 (PMC7897886; doi:10.1186/s12909-021-02562-6)
Supplement: Supplementary file 1 — Additional file 1. Student Survey. [file 12909_2021_2562_MOESM1_ESM.docx]

Medical Student Remote eConsult Participation During the COVID-19 Pandemic

Adam R. Kopp^1^, Sharon Rikin MD^2^, Todd Cassese MD^3^, Matthew A. Berger MD^3^, Amanda C. Raff MD^4^, Inessa Gendlina MD PhD^5*^

(1) Albert Einstein College of Medicine, 1300 Morris Park Avenue, Bronx, New York 10461, USA

(2) Department of Medicine, Division of General Internal Medicine, Albert Einstein College of Medicine and Montefiore Medical Center, 1300 Morris Park Avenue, Bronx, New York 10461, USA

(3) Department of Medicine, Division of Hospital Medicine, Albert Einstein College of Medicine and Montefiore Medical Center, 1300 Morris Park Avenue, Bronx, New York 10461, USA

(4) Department of Medicine, Division of Nephrology, Albert Einstein College of Medicine and Montefiore Medical Center, 1300 Morris Park Avenue, Bronx, New York 10461, USA

(5) Department of Medicine, Division of Infectious Diseases, Albert Einstein College of Medicine and Montefiore Medical Center, 1300 Morris Park Avenue, Bronx, New York 10461, USA

(*) Corresponding author: igendlin@montefiore.org

**Student Survey:**

A) Background Information

A1. How much time have you spent on an inpatient internal medicine consultation team?

-I have never worked with an inpatient consultation team

-Less than 1 week

-Between 1 week and 1 month

-More than 1 month

A2. How much time have you spent providing care via telehealth?

-I have never provided care via telehealth

-Less than 1 week

-Between 1 week and 1 month

-More than 1 month

B) eConsult experience Multiple Choice Questions

B1. Where were you physically located while preforming eConsults?

-A healthcare setting

-Einstein campus not in a healthcare setting (e.g., office, library)

-Einstein housing

-Off-campus residence

-In multiple locations

-Other (please specify)

B2. How many weeks in total did you spend participating in eConsults?

-Less than 1

-1

-2

-3

-4

-5

-More than 5

B3. Rank the following in terms of their benefit to you while rotating on the eConsult service. Rank the most beneficial as “1” to the least beneficial as “12.”

-I consulted on patients who had a wide range of pathology

-I learned how to manage COVID-19 and related conditions

-I worked collaboratively with the assigned attending physician

-I learned about the field of the eConsult team

-I had autonomy in being the first person to work-up a patient

-I developed better understanding of telehealth

-I developed a better understanding of eConsults

-I improved in my oral presentation skills

-I improved in my note writing skills

-I learned how to effectively communicate with a primary team

-I feel more comfortable providing clinical recommendation

-I feel more comfortable making clinical decisions

B4. Which of the following do you believe were challenges related to rotating on the eConsult service? (check all that apply)

-There was not enough diversity of pathology

-My role in patient care was unclear to me

-The service was too busy to allow enough teaching

-The workload was inconsistent, sometimes being too much and other times being too little

-I missed the experience of working on a live consultation service

-I missed getting to see patients

-It was too difficult to control my schedule and time

-I was easily distracted when working remotely on eConsults

-I had too many competing priorities to fully engage with eConsults

-I did not have the expertise to function the way I would have liked

-Other (please specify)

B5. The “art of medicine,” good doctoring, empathy, kindness, compassion and intrinsic ethical behavior are aspects of medicine that are often role-modeled (both negatively and positively) to medical students by residents, fellows and attendings. Was your experience of “role-modeling” different on the eConsult service as compared to other live medical service experiences?

-Yes

-No

-Unsure

B6. Role-modeling on the eConsult service by attendings was primarily:

-Positive

-Negative

-Unsure

B7. The learning environment for eConsults was overall:

-Positive

-Negative

-Unsure

B8. In your opinion, what is the ideal make-up of a clerkship in the future?

-Live patient experiences only

-A mix of live patient experiences and some outpatient telehealth (video or telephone calls to patients)

-A mix of live patient experiences and some inpatient eConsults

-Other (please specify)

B9. Did your experience on internal medicine subspecialty eConsults make you more or less likely to choose internal medicine as a career?

-More

-No effect

-Less

B10. Did your experience on subspecialty eConsult service make you more or less likely to choose that subspecialty as a career?

-More

-No effect

-Less

B11. Would you recommend an eConsult experience to other students?

-Yes

-No

-Unsure

B11b. Why or why not?

B12. When recommending novel treatments for patients, which sources did you use to formulate your clinical decision making? (check all that apply)

-Other team members (e.g., attendings, fellows)

-Primary literature

-News reports

-Institutional guidelines (Montefiore)

-Institutional guidelines (outside hospitals)

-Expert-based society guidelines (e.g., IDSA, ASN)

-National governmental guidelines

-Point-of-care medical resources (e.g., UpToDate)

-Other (please specify)

C) Likert Scale Questions

*Please answer the following with ‘Strongly Agree,’ ‘Agree,’ ‘Disagree,’ ‘Strongly Disagree’*

C1. This experience helped to expand my knowledge about the role of a consultant

C2. This experience helped to expand my knowledge about management of COVID-19

C3. This experience provided opportunities to improve my clinical reasoning

C4. I was provided with clear objectives and expectations

C5. I was provided with a clear explanation of my role on the team

C6. Attendings promoted interactive and engaged learning

C7. This experience helped me to think critically about the evidence for the use of novel treatments

C8. I would participate in a remote, eConsult experience again

C9. Participation had a positive effect on my well being

D) Short Answer Questions

*Please answer the following with as much detail as possible.*

D1. What do you feel were the greatest strengths of this program?

D2. What do you feel were the greatest limitations of this program?

D3. What recommendations do you have to improve this program? Please comment specifically on any changes to infrastructure and operations.

D4. How were you supervised during this experience (e.g., how did you contact your attending, how often did you speak to your attending, how were you assigned consults)?

D5. What resources did you use to facilitate learning during this experience?

D6. Please comment on the experience of providing patient care without actual patient contact.

D7. What was your motivation for participating in this program?

D8. What training did you have prior to starting this experience and what additional training would have been beneficial to you?
