# Supplementary material for: Medical student remote eConsult participation during the COVID-19 pandemic
Source: BMC Med Educ. 2021 Feb 22;21:120. doi: 10.1186/s12909-021-02562-6 (PMC7897886; doi:10.1186/s12909-021-02562-6)
Supplement: Supplementary file 2 — Additional file 2. Faculty Survey. [file 12909_2021_2562_MOESM2_ESM.docx]

Medical Student Remote eConsult Participation During the COVID-19 Pandemic

Adam R. Kopp^1^, Sharon Rikin MD^2^, Todd Cassese MD^3^, Matthew A. Berger MD^3^, Amanda C. Raff MD^4^, Inessa Gendlina MD PhD^5*^

(1) Albert Einstein College of Medicine, 1300 Morris Park Avenue, Bronx, New York 10461, USA

(2) Department of Medicine, Division of General Internal Medicine, Albert Einstein College of Medicine and Montefiore Medical Center, 1300 Morris Park Avenue, Bronx, New York 10461, USA

(3) Department of Medicine, Division of Hospital Medicine, Albert Einstein College of Medicine and Montefiore Medical Center, 1300 Morris Park Avenue, Bronx, New York 10461, USA

(4) Department of Medicine, Division of Nephrology, Albert Einstein College of Medicine and Montefiore Medical Center, 1300 Morris Park Avenue, Bronx, New York 10461, USA

(5) Department of Medicine, Division of Infectious Diseases, Albert Einstein College of Medicine and Montefiore Medical Center, 1300 Morris Park Avenue, Bronx, New York 10461, USA

(*) Corresponding author: igendlin@montefiore.org

**Faculty Survey:**

A) Likert Scale Questions

*Please answer the following with ‘Strongly Agree,’ ‘Agree,’ ‘Disagree,’ ‘Strongly Disagree’*

A1. Student involvement had a positive impact on patient care

A2. Student involvement had a positive impact on my well-being

A3. Student involvement was detrimental to work productivity

A4. Student involvement increased my satisfaction with performing eConsults

A5. I was able to provide high quality teaching to the student on my team

A6. I would be willing to have a student on my eConsult team again in the future

A7. Student involvement resulted in a more academic environment on the team

A8. It is important that medical schools teach students about telehealth

A9. It is important that medical schools teach students about e consultation.

B) Short Answer Questions:

*Please answer the following with as much detail as possible.*

B1. What do you feel were the greatest strengths of this program?

B2. What do you feel were the greatest limitations of this program?

B3. What recommendations do you have to improve this program?

B4. What are some of the key principles of good eConsults involving medical student?
